# Mesenchymal stem cell-derived extracellular vesicles transfer miR-598 to inhibit the growth and metastasis of non-small-cell lung cancer by targeting THBS2

**DOI:** 10.1038/s41420-022-01283-z

**Published:** 2023-01-06

**Authors:** Xuebo Li, Fan Wu

**Affiliations:** 1 Department of General Medicine, The Fourth Affiliated Hospital of China Medical University, 110032 Shenyang, P. R. China; 2 Department of Psychiatry, The Fourth Affiliated Hospital of China Medical University, 110032 Shenyang, P. R. China

**Keywords:** Non-small-cell lung cancer, Non-small-cell lung cancer

## Abstract

Non-small-cell lung cancer (NSCLC) is the subtype of lung cancer, which accounts for about 85% of diagnosed lung cancer cases, and is without any effective therapy. Emerging evidence has revealed microRNA-598 (miR-598) as potential therapeutic target and diagnostic marker of NSCLC. In the present study, we sought to define the role of mesenchymal stem cells (MSCs)-derived extracellular vesicles (EVs) containing miR-598 in NSCLC. Co-culture experiments were conducted to examine the secretion of miR-598 by MSCs and the uptake of EVs by NSCLC cells. The expression of miR-598 in NSCLC cell lines, tissues, and MSC-derived EVs was detected by the RT-qPCR. After treatment with MSCs-EVs, CCK-8 and Transwell assays were adopted to evaluate the effects of miR-598 on proliferation, migration, and invasion capacities of NSCLC cells. Finally, the effects of miR-598 on tumor growth and metastasis were further validated in vivo through subcutaneous tumorigenesis and experimental pulmonary metastasis in nude mice. We found that MSCs-derived EVs could deliver miR-598 into NSCLC cells, where miR-598 specifically targeted and bound with mRNA of THBS2 to inhibit its translational process. By suppressing the promoting effects of THBS2 on the proliferation, migration, and invasion of NSCLC cells, the EV treatment reduced the progression of NSCLC. Notably, these inhibitory effects were reversed by concomitantly overexpressing THBS2. Overall, we find that MSCs-derived EVs containing miR-598 targets THBS2 to inhibit the proliferation and migration of NSCLC cells in vivo and in vitro.

## Introduction

Lung cancer still ranks as the second most common cancer worldwide, rendering it a leading cause of cancer death. According to recent statistics, approximately two million cases of lung cancer are diagnosed worldwide each year, and the disease claims the lives of over 75% of those affected [1]. According to the features of the cancer cells, lung cancer is generally classified into two subtypes: non-small-cell lung cancer (NSCLC) and small-cell lung cancer (SCLC), where which NSCLC is more widespread than SCLC and accounts for approximately 85% of diagnosed lung cancer cases [2]. The association between smoking and lung cancer has long been established and verified [3]; however, about 25% of cases occur in non-smokers worldwide, and this proportional incidence is even higher in Asia [4]. Recent studies have presented that, besides smoking, air pollution, including exposure to kitchen lampblack, emerges as a non-negligible risk factor [5]. In the recent years, novel non-surgical treatments for different subtypes of NSCLC, such as chemotherapies and immunotherapies, are emerging, largely owing to the elucidation of molecular mechanisms of the disease [6]. Thus, more efforts should be concentrated on the investigation of the regulatory pathways of NSCLC to enable the development of new-generation therapeutic strategies [7].

MicroRNAs (miRs or miRNAs), members of a class of small non-coding RNA, have been extensively demonstrated to play significant roles in the developmental pathways of multiple types of cancer [8]. Extracellular vesicles (EVs) secreted by stem cells, including mesenchymal stem cells (MSCs), are important carriers of miRNAs for promoting efficient intercellular delivery [9]. miR-598 has been regarded as a tumor suppressor in different types of cancer, including osteosarcoma [10], colorectal cancer [11], and gastric cancer [12]. More importantly, miR-598 has been demonstrated to suppress cell proliferation, migration, and invasion capabilities in NSCLC by down-regulating Derlin-1 and epithelial-mesenchymal transition [13], or the zinc finger E-box-binding homeobox 2 (ZEB2) [14]. The bioinformatic analysis in the current study revealed the targeting relationship between miR-598 and Thrombospondin-2 (THBS2), a disulfide-linked homotrimeric glycoprotein that mediates cell-to-cell and cell-to-matrix interactions. THBS2 has been reported to be dysregulated in numerous cancers, including NSCLC [15]. Moreover, THBS2 demonstrated the ability to alter the biological characteristics of cancer cells to regulate the progression of cancer [16]. However, the specific function of THBS2 in NSCLC s remains to be discovered. In this study, we therefore constructed both cellular and animal models to systematically investigate the regulating pathways initiated by miR-598 in NSCLC.

## Results

### miR-598 is poorly expressed in NSCLC and predictive of poor prognosis of NSCLC patients

**Fig. 1:**
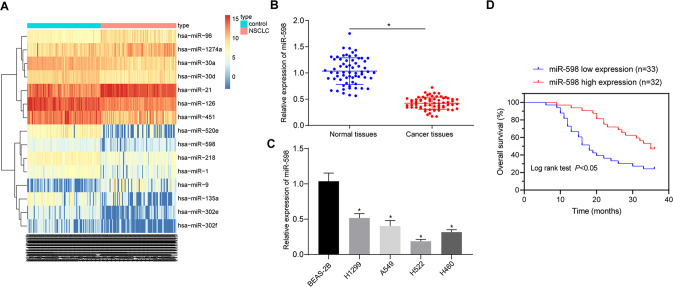
Expression of miR-598 in NSCLC and its correlation with patients’ prognosis. **A** Heat map of the GSE102286 dataset; the ordinate indicates the name of the differential gene, the histogram at the upper right is the color scale, and the color changes from top to bottom indicate the expression value in the dataset data from large to small; each rectangle in the graph corresponds to the expression value of a sample, and each column represents the expression level of all genes in each sample; the left dendrogram represents the cluster analysis results of different genes from different samples; the top bar represents the sample type, and the box at the upper right represents the sample color reference, in which, blue is the normal control sample, while red is the tumor sample. **B** Expression of miR-598 in the tumor and adjacent normal tissues of NSCLC patients by RT-qPCR; *n* = 65; *p < 0.05 vs. adjacent normal tissues. **C** Expression of miR-598 in four NSCLC cell lines (H1299, A549, H522, and H460) and one non-tumorigenic bronchial epithelium BEAS-2B cell line by RT-qPCR; *p < 0.05 vs. BEAS-2B cell line. **D** Correlation between miR-598 expression and prognosis of NSCLC patients by Kaplan-Meier survival analysis. Data between the tumor tissues and adjacent normal tissues were compared by paired *t* test. Data among mul*t*iple groups were compared by ANOVA with Tukey’s post hoc test. Cell experiments were repeated three times.

A R language program was used for the differential analysis of the NSCLC datasets; 43 differentially expressed genes were selected from the miRNA GSE102286 dataset based on the following criteria: |log_2_FC| > 1.5 and adj.P.Val < 0.05. A heat map was plotted for the top 15 differentially expressed miRNAs of the GSE102286 dataset, the results of which depicted poor miR-598 expression in NSCLC tumor tissues compared to normal tissues (Fig. 1A). Reverse transcription quantitative chain reaction (RT-qPCR) results indicated that the miR-598 expression was decreased in NSCLC tissues relative to normal para-cancerous tissues (Fig. 1B). Similarly, the miR-598 expression in NSCLC cell lines (H1299, A549, H522, and H460) was lower than that in the non-tumorigenic bronchial epithelium BEAS-2B cell line (Fig. 1C), with a moderate level of expression being found in A549 and H460 cell lines, which were selected for subsequent cell experiments. In addition, Kaplan-Meier survival analysis of clinical data revealed that the NSCLC patients with lower expression of miR-598 presented with worse prognosis (Fig. 1D). These results suggested that miR-598 is poorly expressed in NSCLC, which may be positively related with poor prognosis of NSCLC patients.

### MSCs-EVs transfer miR-598 into NSCLC cells

**Fig. 2:**
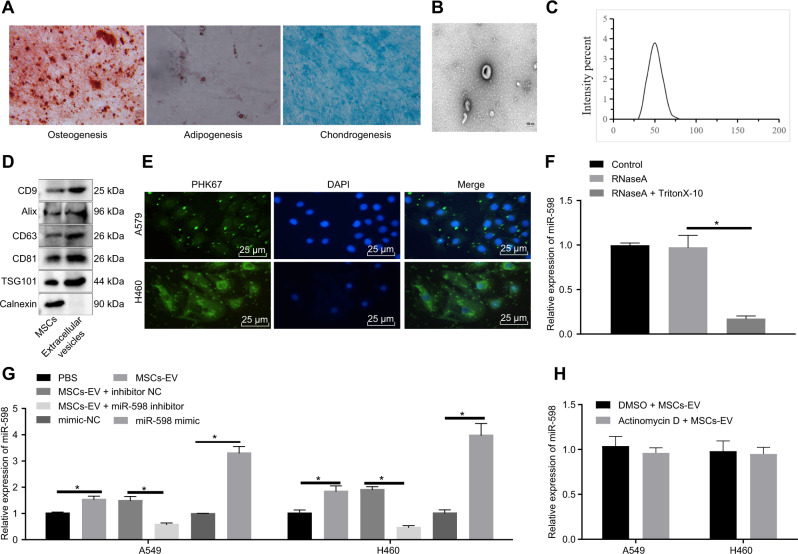
Characterization of MSCs and MSCs-EVs and uptake of MSCs-EVs by NSCLC cells. **A** Identification of the osteogenic differentiation by Alizarin red S staininge, adipogenic differentiation by Oil red O staining, and chondrogenic differentiation by Alcian Blue staining. **B** Morphology of MSCs-EVs observed by TEM. **C** Size distribution of MSCs-EVs measure by DLS. **D** Specific surface marker proteins of MSCs-EVs detected by Western blot analysis. **E** Uptake and internalization of PKH67-labeled MSCs-EVs by the cytoplasm of A549 and H460 cells observed under fluorescence microscope. **F** Expression of miR-598 in A549 and H460 cells after treatment with RNase A and Triton X-100 by RT-qPCR. **G** Expression of miR-598 in A549 and H460 cells after different treatments by RT-qPCR. H Expression of miR-598 in A549 and H460 cells treated with MSCs-EVs after actinomycin D treatment by RT-qPCR. * *p* < 0.05 between two groups. Data between two groups were compared by an unpaired *t* test. Da*t*a among multiple groups were compared by ANOVA with Tukey’s post hoc test. Cell experiments were repeated three times.

An ever-increasing body of evidence that MSCs can mediate antitumor activity and inhibit tumor growth [17, 18]. Therefore, MSCs were isolated from human bone marrows and flow cytometry showed that: CD73 (97%), CD90 (99%), CD105 (96%) were highly expressed, while CD19 (5%), CD45 (4%), CD34 (5%), CD14 (3%), and HLA-DR (5%) were poorly expressed (Supplementary Fig. 1). In addition, the differentiation capacity of MSCs were evaluated, revealing that the isolated and cultured cells had osteogenic, adipogenic, and chondrogenic differentiation capabilities (Fig. 2A), confirming the successful isolation of MSCs.

Human bone marrow MSCs (hBMSCs) have been found to have a high capacity to secret EVs, and EVs-carried miRNAs can be used as predictive biomarkers for lung cancer [19, 20]. Therefore, to further investigate the function of EVs-carried miRNAs in NSCLC, we isolated and characterized EVs from the incubation medium of MSCs. TEM observation indicated that MSCs-EVs were of cup-like or spherical shape (Fig. 2B). A DLS test revealed that the diameters of MSCs-EVs were mainly distributed in the range of 40–150 nm (Fig. 2C). Western blot analysis of EV surface markers suggested that, compared with cell lysates, the expression of CD63, CD81, CD9, Alix and TSG101 was increased in MSCs-EVs (Fig. 2D), indicating that MSCs-EVs were successfully isolated.

To determine the internalization ability of A549 and H460 NSCLC cell lines towards MSCs-EVs, MSCs-EVs were labeled with green fluorescent dye (PKH67) for 4 h. The A549 and H460 cells showed a strong cytoplasmic green fluorescence signal under a fluorescence microscope (Fig. 2E), indicating that the A549 and H460 cells could internalize MSCs-EVs and their contents. Next, aiming to further confirm that miR-598 could be transferred into A549 and H460 cells by EVs, we extracted RNA from the conditioned medium of MSCs treated with RNase A. As RT-qPCR suggested, there were no obvious changes in the miR-598 expression in the RNase A-treated cells relative to the control cells. However, the cells treated with RNase A combined with Triton X-100 exhibited a decreased miR-598 expression (Fig. 2F). Moreover, this experiment showed that the miR-598 expression was elevated in A549 and H460 cells following MSCs-EVs treatment relative to those following PBS treatment, while miR-598 mimic treatment increased the miR-598 expression in A549 and H460 cells relative to mimic negative control (NC) treatment. In MSCs-EVs-treated cells exposed to miR-598 inhibitor, miR-598 expression was down-regulated relative to those treated with inhibitor NC (Fig. 2G). To further rule out the possibility that the miR-598 expression was induced endogenously, A549 and H460 cells co-incubated with MSCs-EVs were treated with the RNA synsthesis inhibitor actinomycin D. As shown in Fig. 2H, there was no significant difference between the miR-598 expression in actinomycin D-treated cells and untreated cells, indicating that the miR-598 expression is not induced endogenously. In summary, our data presented that miR-598 is highly expressed in MSCs-EVs, and could be transferred in NSCLC cells by MSCs-EVs in vitro.

### MSCs-EVs transfer miR-598 to inhibit the proliferation, migration, and invasion capabilities of NSCLC cells in vitro

**Fig. 3:**
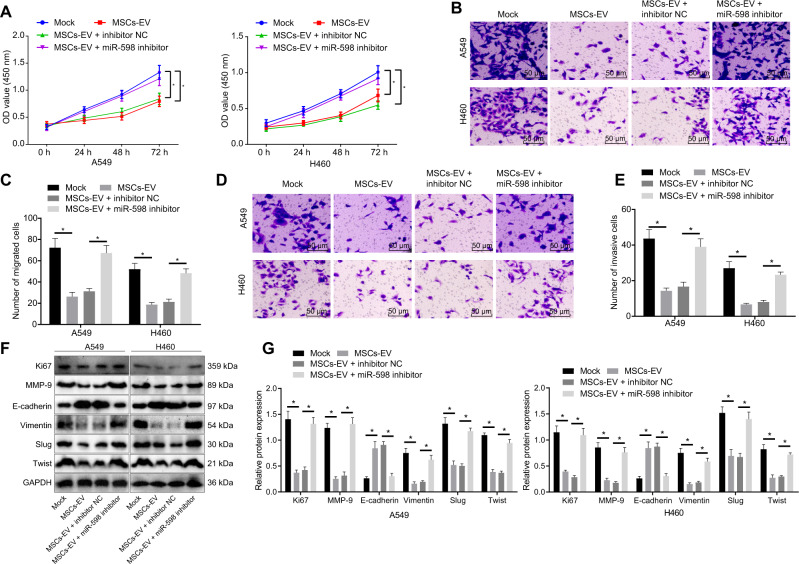
Effects of EVs-derived miR-598 on the proliferation, migration, and invasion abilities of NSCLC cells. **A** Proliferative ability of A549 and H460 cells detected by CCK-8 assay. **B** Migrated ability of A549 and H460 cells detected by Transwell assay. **C** Statistical analysis of (**B**). **D** Invasive ability of A549 and H460 cells detected by Transwell assay. **E** Statistical analysis of (**D**). **F** Protein bands of Ki67, E-cadherin, Vimentin, MMP-9, Slug, and Twist by Western blot analysis. **G** Protein levels of Ki67, E-cadherin, Vimentin, MMP-9, Slug, and Twist by Western blot analysis (statistical analysis of (**F**)). *p < 0.05 between two groups. Data between two groups were compared by an unpaired *t* test. Da*t*a among multiple groups were compared by ANOVA with Tukey’s post hoc test. Comparisons among groups at different time points were performed using repeated measures ANOVA with Bonferroni’s post hoc test. Cell experiments were repeated three times.

Studies have shown that miR-598 can inhibit the proliferation and migration capabilities of NSCLC cells [13]. To verify the effects of EVs-derived miR-598 on the biological characteristics of NSCLC cells, we treated A549 and H460 cells with MSCs-EVs, MSCs-EVs + inhibitor NC, or MSCs-EVs + miR-598 inhibitor, with sham-treated cells as a control. CCK-8 and Transwell experiments results indicated that the proliferative, migrated, and invasive capabilities of the MSCs-EVs-treated cells were reduced when compared with that of the sham-treated cells. However, these cababilites were promoted in the cells treated with MSCs-EVs + miR-598 inhibitor relative to those following MSCs-EVs + inhibitor NC treatment (Fig. 3A–E). These data suggested that miR-598 inhibitor could reduce the inhibitory effects of MSCs-EVs on proliferation, migration, and invasion of A549 and H460 cells. In addition, Western blot analysis confirmed that the protein levels of proliferation-related factor Ki67, Vimentin, matrix metalloproteinase-9 (MMP-9), zinc-finger protein Slug, and basic helix-loop-helix protein Twist were reduced, whereas the E-cadherin protein level was increased in the MSCs-EVs-treated cells when compared with the sham-treated cells. As compared to the cells treated with MSCs-EVs and inhibitor NC, the cells treated with MSCs-EVs and miR-598 inhibitor showed increased protein levels of Ki67, Vimentin, MMP-9, Slug, and Twist, as well as decreased E-cadherin protein level (Fig. 3F, G). Taken together, these results demonstrated that EVs-carried miR-598 inhibited the proliferation, migration, and invasion capabilities of NSCLC cells.

### MSCs-EVs transfer miR-598 to inhibit the tumor growth and metastasis of NSCLC in vivo

**Fig. 4:**
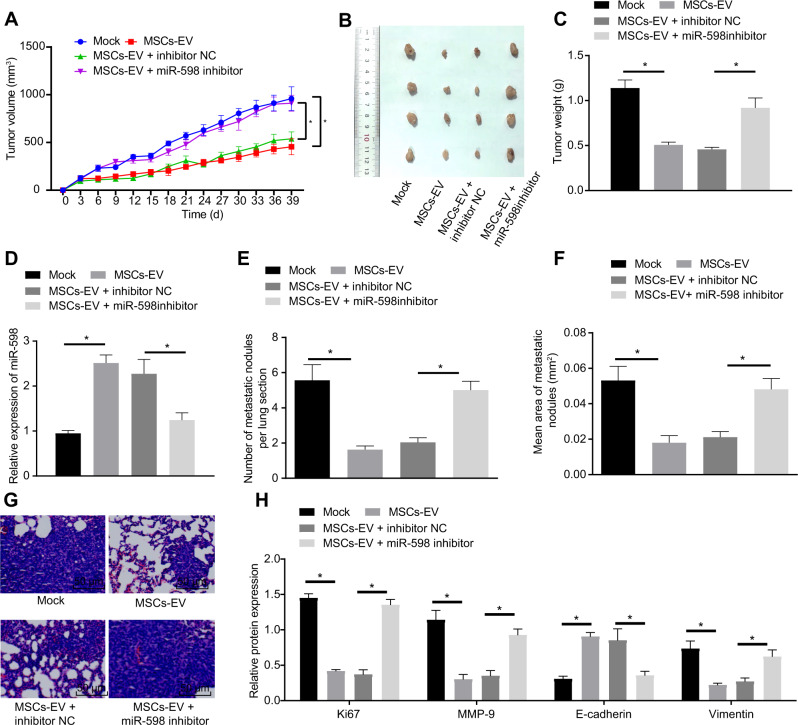
Effects of EVs-derived miR-598 on tumor growth and metastasis of NSCLC in vivo. **A** Tumor volume in the nude mice after different treatments. **B** Representative white-light images of tumors after different treatment. **C** Tumor weight in the nude mice after different treatments. **D** Expression of miR-598 in tumors of the nude mice after different treatments by RT-qPCR. **E** Number of metastatic nodules in the lungs of nude mice with pulmonary metastasis. **F** Area of metastatic nodules in the lungs of nude mice. **G** Morphology of metastatic nodules in the lungs of nude mice e observed by H&E staining. **H** Protein levels of Ki67, E-cadherin, Vimentin, and MMP-9 in metastatic nodules in the lungs of nude mice by Western blot analysis. *n* = 6 in each group in the subcutaneous tumorigenesis experiments, *n* = 15 in each group in the pulmonary metastasis experiments. *p < 0.05 between two groups. Data among multiple groups were compared by ANOVA with Tukey’s post hoc test. Comparisons among groups at different time points were performed using repeated measures ANOVA with Bonferroni’s post hoc test.

To study the effects of miR-598 transferred by MSCs-EVs on the NSCLC development in vivo, we constructed a subcutaneous tumorigenesis model in nude mice. Results showed that the final volume and weight of tumors were decreased in the mice following treatment of MSCs-EVs when compared with untreated mice, while MSCs-EVs + miR-598 inhibitor treatment increased the volume and weight of tumors relative to the MSCs-EVs + inhibitor NC treatment (Fig. 4A–C). Moreover, RT-qPCR revealed that the miR-598 expression in the tumors of MSCs-EVs-treated mice was higher than that of the untreated mice, which in the tumors of MSCs-EVs + miR-598 inhibitor-treated mice was suppressed when compared to that of the MSCs-EVs + inhibitor NC-treated mice (Fig. 4D). Western blot analysis revealed that the Ki67 protein level in the tumors of nude mice was reduced by MSCs-EV treatment, which was promoted by additional miR-598 inhibitor treatment (Fig. 4H). In addition, we established a pulmonary metastasis model and extracted the lungs for examination after various treatments. MSCs-EV treatment reduced the number and size of metastatic nodules in the lungs, which were increased by additional miR-598 inhibitor treatment (Fig. 4E–G). Then, as Western blot analysis detected, the protein levels of Vimentin and MMP-9 were reduced in the MSCs-EVs-treated mice relative to untreated mice, while the expression of E-cadherin was enhanced; these effects were reversed by miR-598 inhibitor treatment (Fig. 4H). Meanwhile, similar results were obtained in considering the roles of MSCs-EVs secreted miR-598 on the tumorigenesis and metastasis of H460 cells in nude mice, as reported in Supplementary Fig. 2. To summarize, these in vivo experiments confirmed that EVs-carried miR-598 inhibited the tumor growth and metastasis of NSCLC.

### THBS2 is a direct target gene of miR-598

**Fig. 5:**
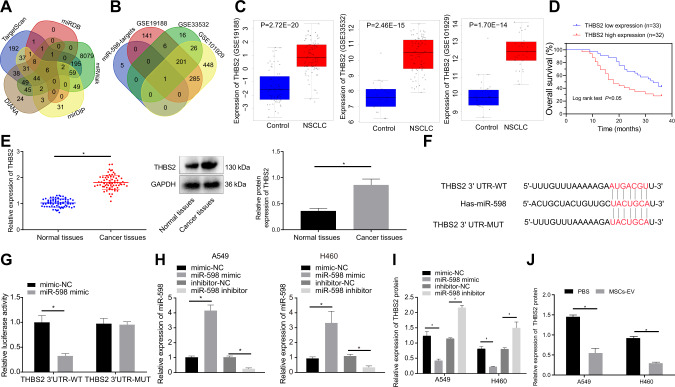
Relationship between THBS2 and miR-598. **A** Intersection of the miR-598 target genes predicted by TargetScan, miRDB, miRWalk, mirDIP, and DIANA databases. **B** Intersection of the differentially expressed genes in GSE19188, GSE33532, and GSE101929 datasets and the miR-598 target genes. **C** THBS2 expression in the GSE19188, GSE33532, and GSE101929 datasets. **D** Correlation between THBS2 expression and prognosis of NSCLC patients by Kaplan–Meier analysis. **E** The mRNA and protein expressions of THBS2 in tumor tissues and adjacent normal tissues of NSCLC by RT-qPCR and Western blot analyses. **F** The specific binding sites between miR-598 and THBS2 predicted online (Targetscan). **G** Targeting relationship between miR-598 and THBS2 verified by dual luciferase reporter assay. **H** Expression of miR-598 in A549 and H460 cells after different transfections by RT-qPCR. **I** THBS2 protein level in A549 and H460 cells after different transfections by Western blot analysis. **J** THBS2 protein level in A549 and H460 cells treated with MSCs-EVs by Western blot analysis. *p < 0.05 between two groups. Data between tumor tissues and adjacent normal tissues were compared by paired *t* test. Data between two groups were compared by unpaired *t* test. Cell experiments were repeated three times.

The above experiments revealed that EVs-encapsulated miR-598 inhibited the proliferation, migration, and invasion capabilities of NSCLC cells, in vitro and in vivo. To further investigate the downstream regulatory mechanism of miR-598, we predicted the target genes of miR-598 based on TargetScan, miRDB, miRWalk, mirDIP, and DIANA databases. As shown in Fig. 5A, we obtained 665, 20, 8531, 273, and 252 target genes from the aforementioned online prediction databases respectively Venn analysis indicated six intersected genes (SLC6A13, MYH8, SLC35D3, PANK1, THBS2, and MN1), which were regarded as the target genes of miR-598. Differential analysis of the NSCLC gene datasets screened 634, 250, and 961 differential genes from GSE19188, GSE33532, and GSE101929 datasets, respectively. After intersection of these sets of differentially expressed genes, we isolated six miR-598 target genes, and finally obtained THBS2 gene by Venn analysis (Fig. 5B). The findings for expression of THBS2 in GSE19188, GSE33532, and GSE101929 datasets showed thatTHBS2 had abnormally highly expression in NSCLC (Fig. 5C), leading us to speculate that the differential expression of THBS2 may be regulated by miR-598. Besides, as suggested by Kaplan-Meier survival analysis, NSCLC patients with high expression of THBS2 presented a worse prognosis (Fig. 5D). RT-qPCR and Western blot analysis results showed that, when compared with the adjacent normal tissues, THBS2 was highly expressed in the tumor tissues of NSCLC patients (Fig. 5E).

The specific binding sites between miR-598 and THBS2 were predicted by Targetscan (Fig. 5F). Dual luciferase report assay was then utilized to verify the targeting relationship between miR-598 and THBS2, the results of which indicated that the fluorescence intensity of the miR-598 mimic + THBS2 3′UTR-WT co-transfected cells was decreased, compared with that of the mimic NC + THBS2 3′UTR-WT co-transfected cells, while there was no significant change in fluorescence intensity between the mimic NC + THBS2 3′UTR-MUT co-transfected cells and the miR-598 mimic + THBS2 3′UTR-MUT co-transfected cells (Fig. 5G). In addition, RT-qPCR and Western blot analysis results presented that the expression of miR-598 in the miR-598 mimic-treated cells was increased relative to the mimic NC-treated cells, while the level of THBS2 protein was relatively down-regulated. When compared with inhibitor NC treatment, miR-598 inhibitor treatment decreased the miR-598 expression and increased the THBS2 protein level in cells (Fig. 5H, I). After the treatment of A549 and H460 cells with MSCs-EVs, as displayed in Fig. 5J, Western blot analysis indicated that the intracellular THBS2 protein level was reduced relative to that in the PBS-treated cells. From the above data, we can conclude that THBS2 is a downstream target gene of miR-598, and could be negatively regulated by miR-598.

### THBS2 promotes the proliferation, migration, and invasion of NSCLC cells

**Fig. 6:**
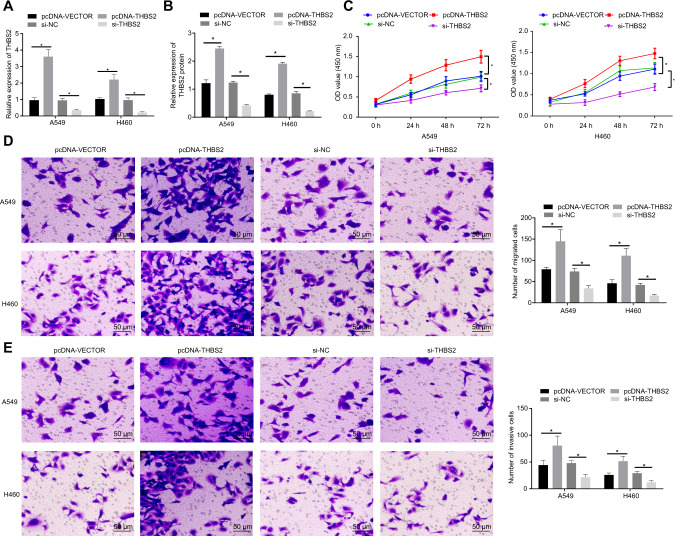
Effects of THBS2 on the proliferation, migration, and invasion abilities of NSCLC cells. **A** THBS2 mRNA expression in A549 and H460 cells treated with pcDNA-THBS2 or si-THBS2 by RT-qPCR. **B** THBS2 protein level in A549 and H460 cells treated with pcDNA-THBS2 or si-THBS2 by Western blot analysis. **C** Proliferative ability of A549 and H460 cells treated with pcDNA-THBS2 or si-THBS2 detected by CCK-8 assay. **D** Migration ability of A549 and H460 cells treated with pcDNA-THBS2 or si-THBS2 detected by Transwell assay. **E** Invasive ability of A549 and H460 cells treated with pcDNA-THBS2 or si-THBS2 detected by Transwell assay. *p < 0.05 between two groups. Data among multiple groups were compared by ANOVA with Tukey’s post hoc test. Comparisons among groups at different time points were performed using repeated measures ANOVA with Bonferroni’s post hoc test. Cell experiments were repeated three times.

To verify the effects of THBS2 on the biological functions of NSCLC cells, THBS2 was overexpressed or knocked down in A549 and H460 cells. First, were detected the mRNA and protein expressions of THBS2 in the cells eby RT-qPCR and Western blot analysis (Fig. 6A, B). Results indicated that the expression of THBS2 was increased upon pcDNA-THBS2 treatment and decreased after si-THBS2 treatment. Next, CCK-8 and Transwell assay results showed that the proliferation, migration, and invasion abilities of A549 and H460 cells were promoted by THBS2 overexpression but suppressed by THBS2 knockdown (Fig. 6C–E). Taken together, the above data proved that THBS2 promoted the proliferation, migration, and invasion capabilities of NSCLC cells.

### MSCs-EVs-derived miR-598 down-regulates THBS2 to inhibit the proliferation, migration, and invasion capabilities of NSCLC cells in vitro

**Fig. 7:**
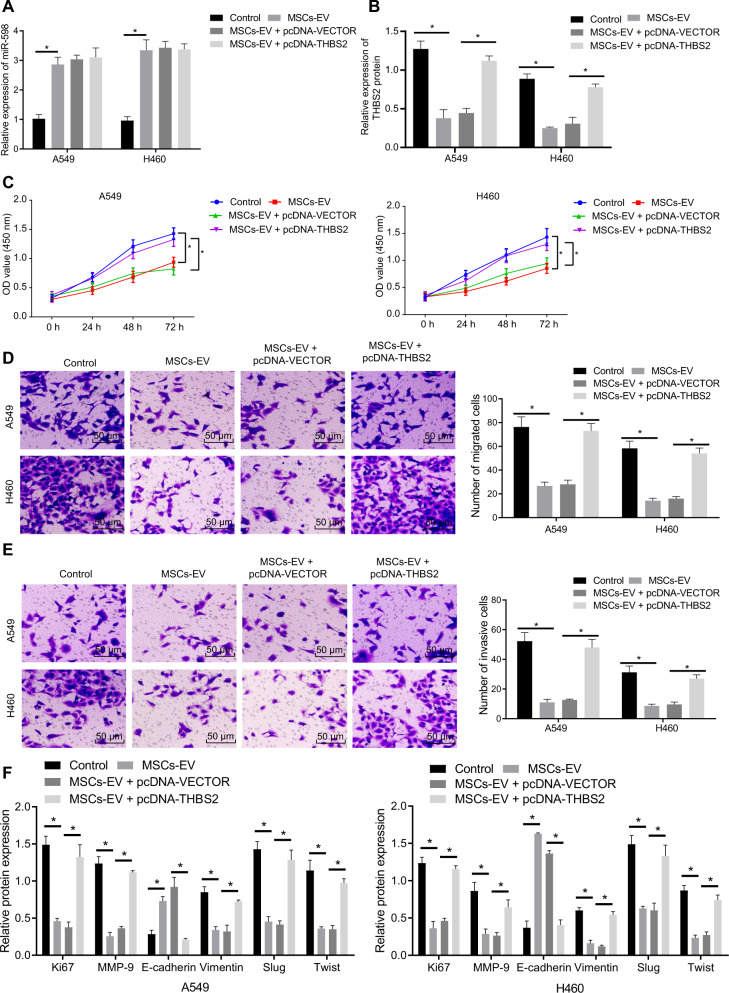
Effects of EVs-derived miR-598 on the proliferation, migration, and invasion abilities of NSCLC cells via targeting of THBS2. **A** Expression of miR-598 in A549 and H460 cells treated with MSCs-EVs and pcDNA-THBS2 by RT-qPCR. **B** THBS2 protein level in A549 and H460 cells treated with MSCs-EVs and pcDNA-THBS2 by Western blot analysis. **C** Proliferative ability of A549 and H460 cells treated with MSCs-EVs and pcDNA-THBS2 detected by CCK-8 assay. **D** Migrated ability of A549 and H460 cells treated with MSCs-EVs and pcDNA-THBS2 detected by Transwell assay. **E** Invasive ability of A549 and H460 cells treated with MSCs-EVs and pcDNA-THBS2 detected by Transwell assay. **F** Protein levels of Ki67, E-cadherin, Vimentin, MMP-9, Slug, and Twist by Western blot analysis. *p < 0.05 between two groups. Comparisons among groups at different time points were performed using repeated measures ANOVA with Bonferroni’s post hoc test. Cell experiments were repeated three times.

To further explore the effects of miR-598 transferred by MSCs-EVs on the biological functions of NSCLC cells by targeting THBS2, we treated A549 and H460 cells with MSCs-EVs and pcDNA-THBS2 plasmid. RT-qPCR showed that the expression of miR-598 was increased in the A549 and H460 cells after this MSCs-EV treatment, while pcDNA-THBS2 plasmid had no effect on the expression of miR-598 (Fig. 7A). Western blot analysis further revealed that MSCs-EVs treatment inhibited the protein level of THBS2 in the cells, while co-treatment with MSCs-EVs and pcDNA-THBS2 enhanced the protein level of THBS2 (Fig. 7B). CCK-8 and Transwell experimental results displayed that MSCs-EVs inhibited the proliferative, migration and invasive capabilities of NSCLC cells, which, conversely, were promoted by overexpressing THBS2 (Fig. 7C–E). Besides, Western blot analysis revealed that, compared with the untreated cells, the expression of Ki67, Vimentin, MMP-9, Slug, and Twist was reduced in the MSCs-EVs-treated cells, while the expression of E-cadherin was increased. When compared with the MSCs-EVs + pcDNA-vector treatment, the MSCs-EVs + pcDNA-THBS treatment was observe to increase the expression of Ki67, Vimentin, MMP-9, Slug, and Twist, and decrease the expression of E-cadherin (Fig. 7F). In brief, all the above experimental data support the hypothesis that EVs-encapsulated miR-598 targets THBS2 to inhibit the proliferation, migration, and invasion capabilities of NSCLC cells in vitro.

### MSCs-EVs-derived miR-598 targets THBS2 to inhibit the tumor growth and metastasis of NSCLC in vivo

**Fig. 8:**
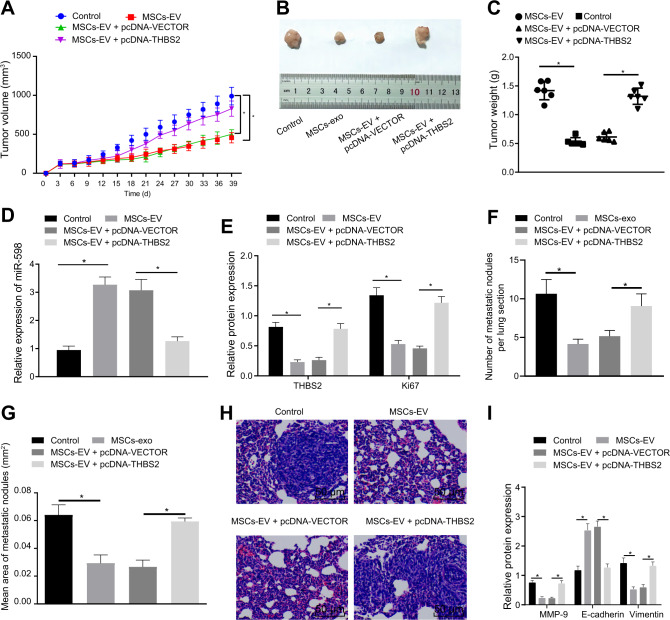
Effects of EVs-derived miR-598 on tumor growth and metastasis of NSCLC in vivo by targeting THBS2. **A** Tumor volume in the nude mice after different treatments. **B** Representative white-light images of tumors after different treatment. **C** Tumor weight in the nude mice after different treatment. **D** Expression of miR-598 in tumors of the nude mice after different treatments by RT-qPCR. **E** Protein levels of THNS2 and Ki67 in tumors of the nude mice measured by Western blot analysis. **F** Number of metastatic nodules in the lungs of nude mice. **G** Area of metastatic nodules in the lungs of nude mice. **H** Morphology of metastatic nodules in the lungs of nude mice observed by H&E staining. **I** Protein levels of E-cadherin, Vimentin, and MMP-9 in metastatic nodules in the lungs of nude mice by Western blot analysis. *n* = 6 in each group in the subcutaneous tumorigenesis experiments, *n* = 15 in each group in the pulmonary metastasis experiments. *p < 0.05 between two groups. Data among multiple groups were compared by ANOVA with Tukey’s post hoc test. Comparison among groups at different time points was performed using repeated measures of ANOVA with Bonferroni’s post hoc test.

To verify whether THBS2 is involved in the inhibitor effects of MSCs-EVs-encapsulated miR-598 on the tumor growth and metastasis of NSCLC, we conducted subcutaneous tumorigenesis experiments in nude mice. The results showed that the final tumor volume and weight in the MSCs-EVs-treated mice were decreased compared with these endpoints in the untreated mice. In MSCs-EVs + pcDNA-THBS2-treated mice, increased tumor volume and weight were observed relative to MSCs-EVs + pcDNA-vector-treated mice (Fig. 8A–C). Then, RT-qPCR results demonstrated that the expression of miR-598 in MSCs-EVs-treated mice was higher than in the untreated mice (Fig. 8D). Western blot analysis revealed reduced protein levels of THBS2 and Ki67 in the MSCs-EVs-treated mice compared with untreated mice, while mice treated with MSCs-EVs + pcDNA-THBS2 showed elevated protein levels of THBS2 and Ki67 relative to those treated with MSCs-EVs + pcDNA-vector (Fig. 8E).

Furthermore, we attempted to investigatee whether miR-598 secreted by MSCs-EVs influenced the tumor metastasis of NSCLC function through the involvement of THBS2. Here, we established a pulmonary metastasis model in nude mice. MSCs-EV treatment was observed to reduce the number and size of metastatic nodules in the lungs of these mice compared with untreated mice, while this decline could be reversed by simultaneously overexpressing THBS2 (Fig. 8F–H). In addition, Western blot analysis suggested that MSCs-EVs suppressed the expression of Vimentin and MMP-9 and enhanced the expression of E-cadherin in the metastases, which were reversed by overexpressing THBS2 (Fig. 8I). In summary, these in vivo experiments also verified that MSCs-EVs transferred miR-598 to inhibit the growth and metastasis of NSCLC in vivo by down-regulating THBS2 expression.

## Discussion

Lung cancer reamins one of the most widespread and deadly cancers in today’s society, constituting approximately 12% of all diagnosed cancers worldwide [21]. The most common subtype of lung cancer, NSCLC, accounts for over 80% of all diagnosed lung cancers [22]. Unraveling new therapeutic targets and biomarkers of NSCLC to promotee the development of novel therapeutic strategies is therefore vital for the ongoing fight against lung cancer, which remains a serious problem despite declining smoking rates the world over [23]. In our study, we found that MSCs-derived EVs could deliver miR-598 into NSCLC cells, and that miR-598 specifically targets THBS2 to inhibit its translation process. In addition, we found that MSCs-EVs-derived miR-598 may exert inhibitory effects on the proliferation, migration, and invasion capabilities of NSCLC cells as well as the growth and metastasis of NSCLC tumors through the down-regulation of THBS2 (Supplementary Fig. 3).

Initially, the results of our preliminary bioinformatic analysis revealed a down-regulated expression of miR-598 in NSCLC samples. As previously reported, miR-598 has been widely found to be poorly expressed in NSCLC specimens and cell lines [13, 14, 24]. In order to validate the bioinformatic analysis results and literature findings, we tested clinically obtained NSCLC tumor tissues and commercially purchased NSCLC cell lines in our study. Consistent with the literature findings, our RT-qPCR assay also demonstrated the poor expression of miR-598 in NSCLC tissues and cells. Besides, as suggested by Kaplan-Meier analysis of the clinical data, we identified a negative correlation between miR-598 expression and survival prognosis of NSCLC patients. These results suggest the important involvement of miR-598 in the development and progression of NSCLC.

The EVs secreted by MSCs can carry and deliver DNA, RNA, and protein cargoes to regulate cellular signaling pathways in target cells [25]. EVs are nanospheres with a bilayer membrane that transport miRs cargoes and can thus overcome some of the limitations of lipid and polymeric miR delivery systems [26]. Of note, MSCs-EVs are emerging as important carriers of miRNAs for the efficient treatment of various diseases [27], specifically lung cancer [28]. In this investigation, we discovered that miR-598 was highly expressed in MSCs-EVs; after internalization of the miR-598-containing MSCs-EVs by NSCLC cells, the intracellular level of miR-598 was correspondingly up-regulated, thus revealing that MSCs-EVs could transfer miR-598 into NSCLC cells. Moreover, MSCs-EVs-encapsulated miR-598 was observed to play a suppressive role in the proliferative, migration, and invasive abilities of NSCLC cells. Meanwhile, subcutaneous tumorigenesis and pulmonary metastasis experiments in vivo also confirmed that EVs-derived miR-598 suppressed tumor growth and lung metastasis. In lines with our findings, a recent study unraveled that miR-598 could suppress the proliferation and invasion of NSCLC cells, thus inhibiting the progression of NSCLC [14]. Additionally, miR-598 has long been recognized as a tumor suppressor in many types of cancer, which acts mainly by regulating the proliferation, migration, and invasion capabilities of corresponding cancer cells [29–31]. Thus, one important finding of our investigation is that MSCs-derived EVs can carry the tumor suppressor miR-598 into NSCLC cells to inhibit the tumor development.

Interestingly, previous research has proved that THBS2 was abnormally expressed in NSCLC [32], which was also fully supported by our initial bioinformatic analysis, in which THBS2 was found to be highly expressed in NSCLC samples. On the other hand, bioinformatic searches in different databases predicted that THBS2 was a downstream target gene of miR-598, which was further confirmed by RT-qPCR, Western blot analysis, and dual luciferase reporter assay performed in this study. Mounting evidence indicate that silencing of THBS2 had the ability to inhibit the proliferation, migration, and invasion capabilities of gastric cancer cells [33], in accord with our present findings for NSCLC cells in vitro. Taking these results together, it is highly possible that THBS2 may be targeted by miR-598 to regulate the progression of NSCLC. We have thoroughly tested this hypothesis in cellular and animal models, and confirmed that MSCs-EVs-secreted miR-598 indeed suppresses the proliferation, migration, and invasion capabilities of NSCLC cells by targeting THBS2 in vitro and in vivo.

In summary, our data reveal that MSCs secreted EVs carried and delivered miR-598 into NSCLC cells. Upon delivery, miR-598 targeted and inhibited the expression of THBS2, which inhibited the proliferation, migration, and invasion of NSCLC cells in vitro, while arresting tumor growth and metastasis in vivo. These findings attest to the proposal that miR-598 may prove to serve as a valuable diagnosis biomarker and therapeutic target for NSCLC. However, investigating the expression of miR-598 in the serum and EVs of patients with lung cancer is a matter for future experiments.

## Materials and methods

### Ethics statement

All research procedures were conducted with the approval of the Ethics Committee of The Fourth Affiliated Hospital of China Medical University and in accordance with the *Declaration of Helsinki*. All patients and/or legal guardians have signed the informed consent documentations. All animal experiments were approved by the Animal Ethics Committee of The Fourth Affiliated Hospital of China Medical University. Great efforts were made to minimize the number of animals used in the experiments and their suffering.

### Bioinformatics analysis

NSCLC miRNA expression dataset GSE102286 and gene datasets GSE19188, GSE33532, and GSE101929 were deposited from the Gene Expression Omnibus (GEO) database (https://www.ncbi.nlm.nih.gov/geo/). An R language program was used to perform differential analyses on the datasets. An affy package was adopted for the background correction and normalization of the expression data, while the limma package was used to screen differentially expressed genes or miRNAs. After the correction of expression data, the P value is expressed by adj.P.Val. Genes or miRNAs with |log_2_FoldChange (FC) | > 1.5 and adj.P.Val < 0.05 were regarded as being significantly differentially expressed. A miRNA differential expression heat map was then drawn. The target genes of miR-598 were predicted by TargetScan (http://www.targetscan.org/vert_71/), miRDB (http://www.mirdb.org/), miRWalk (http://mirwalk.umm.uni-heidelberg.de/), mirDIP (http://ophid.utoronto.ca/mirDIP/), and DIANA (http://diana.imis.athena-innovation.gr/DianaTools/index.php?r=microT_CDS/index). The Venn online analysis tool (http://bioinformatics.psb.ugent.be/webtools/Venn/) was used to calculate and draw custom Venn diagrams to compare target gene prediction results and differentially expressed genes, and to screen out those differentially expressed genes that may be regulated by differential miRNAs.

### Collection of clinical samples

A total of 65 cases of primary tissues and adjacent normal tissues were surgically collected from patients with NSCLC from The Fourth Affiliated Hospital of China Medical University between June 2016 and March 2018. The patients received no chemotherapy or radiotherapy prior to their surgical resection. All obtained tissues were snap-frozen in liquid nitrogen and stored at −80 °C until further use.

### Cell culture

Human NSCLC cell lines (H1299, A549, H522, and H460) and a non-tumorigenic bronchial epithelium BEAS-2B cell line were all purchased from the American Type Culture Collection (ATCC; Manassas, VA, USA). NSCLC cell lines were cultured in Dulbecco’s modified Eagle’s medium (DMEM) containing 10% fetal bovine serum (FBS), 100 U/mL penicillin and 100 µg/mL streptomycin (all from Gibco; Thermo Fisher Scientific, Inc., Waltham, MA, USA). BEAS-2B cell line was grown in LHC-9 medium (Gibco; Thermo Fisher Scientific, Inc.). All cell lines were maintained in a humidified atmosphere containing 5% CO_2_ at 37 °C. Besides, 293T cells were purchased from ATCC, which were cultured in DMEM containing 10% FBS.

### Isolation and identification of hBMSCs

The hBMSCs used in this study were isolated from bone marrow aspirates donated by healthy volunteers between the ages of 29 and 83 years in The Fourth Affiliated Hospital of China Medical University. For the aspirates, a 15-gauge needle was inserted and rotated after 2 mL samples of marrow was aspirated, thus minimizing the contamination by peripheral blood. The cells were cultured in a RPMI-1640 medium supplemented with 10% FBS and penicillin/streptomycin and maintained at 37 °C in a humidified atmosphere containing 5% CO_2_ for 48 h. The plates were washed with PBS buffer solution to remove non-adherant cells and the medium was subsequently renewed. When the hBMSCs reached 80–90% confluence, they were trypsinized and prepared for subculture. Afterwards, hBMSCs that were passaged no more than five times were used in the subsequent experiments. The immunophenotypic characterization of hBMSCs was analyzed by flow cytometry assay. In brief, hBMSCs were trypsinized for 2–4 min, washed with PBS buffer solution without magnesium and calcium, and were later blocked with 10% normal goat serum to prevent nonspecific binding. Next, the cells were incubated with a series of human monoclonal primary antibodies labeled with fluorescein isothiocyanate (FITC) dyes, including CD105, CD14, CD19, CD34, CD45, CD73, CD90, and HLA-DR (1:100; BioLegend, San Diego, CA, USA) for 30 min. Subsequently, the cells were washed with PBS buffer solution, resuspended in 10% normal goat serum, and analyzed with a CyAn ADP Flow Cytometer Analyzer (Beckman Coulter, Brea, CA, USA).

### Cell multi-differentiation

For the differentiation of osteogenic cells, hBMSCs were seeded (1 × 10^5^ cells/well) into 6-well culture plates and cultured to allow attachment; the substrate was then switched to an osteogenic medium containing DMEM supplemented with 0.17 mM L-ascorbic acid, 5% FBS solution, 10 mM β-glycerophosphate, 100 nM dexamethasone (Sigma, St Louis, MO, USA), and 1% penicillin/streptomycin. The cells were allowed to culture for 21-28 days with fresh osteogenic medium replaced every 2 days. When the calcium nodules could be visibly observed by light microscopy, the cells were subjected to Alizarin red S staining. The stained cells were then observed by using a light microscope.

For the differentiation of adipocytes, MSCs were seeded (1 × 10^5^ cells/well) into 6-well culture plates and were left to culture to allow attachment; the substrate was then changed to an adipogenic differentiation cocktail comprising of the following: 1 µM rosiglitazone, 1 µM dexamethasone, 0.5 mM 3-isobutyl-1-methyl-xanthine, 10 µg/mL insulin, 0.2 mM indomethacin, and 1% penicillin/streptomycin in DMEM-low glucose supplemented with glutamine and 10% FBS solution. The medium was renewed after 3 days with DMEM-Low glucose containing glutamine, 10% FBS, 1% penicillin–streptomycin, 1 mM rosiglitazone, and 10 mg/mL insulin every other day. The cells were incubated at 37 °C with 5% CO_2_ for 21–28 days and when the lipid droplets were apparently visible under light microscopy, the cells were dyed by Oil Red O diazo dye. The stained cells were then observed under a light microscope.

For the differentiation of chondrogenic cells, MSCs were seeded into a (2 × 10^6^ cells/tube) 15 mL centrifuge tube in a ball-like structure and cultured at 37 °C in 5% CO_2_ for 24 h; the substrate was then switched to a chondrogenic medium containing DMEM (4.5 g/L glucose) supplemented with the following, 100 nM dexamethasone, 0.35 mM proline, 0.17 mM L-ascorbic acid, 1 mM sodium pyruvate, 1% insulin-transferrin-selenium, 10 ng/mL TGFβ-3 (Sigma), and 1% penicillin/streptomycin. Afterwards, the cells were incubated at 37 °C with 5% CO_2_ for 21-28 days with the renewal of fresh medium every 2 days. When the global cells were grown into balls with 1.5–2.0 mm diameter, the cells were sliced into pieces and dyed with Alcian Blue staining. The sections were then observed using a light microscope.

### Isolation of MSCs-EVs

hBMSCs were seeded into 6-well plates at a density of 1 × 10^5^ cells/well, and cultured in a DMEM medium containing 10% FBS solution and 1% Penicillin-Streptomycin (all from Gibco; Thermo Fisher Scientific, Inc., Waltham, MA, USA) until the cells reached 80-90% confluence. hBMSCs were cultured in a RPMI1640 medium containing EV‐depleted FBS (by centrifugation 18 h at 100,000 g) for 48 h. The supernatants were centrifuged at 2000*g* for 10 min and then 10,000*g* for 30 min to isolate the debris and apoptotic bodies (Beckman Avanti Centrifuge J‐26XP, Beckman Coulter, Brea, CA, USA) from the supernatants. Subsequently, the supernatants were centrifuged at 110,000*g* for an additional 70 min (Beckman Optima L‐80 XP Ultracentrifuge with 70Ti rotor), followed by washing with PBS solution and purification by centrifugating at 110,000*g* for 70 min. All the centrifugations were performed at 4 °C. The pellet was resuspended in PBS solution and sterilized by filtration through a 0.22 μm filter (Millipore, Darmstadt, Germany).

### Identification of MSCs-EVs

The size of MSC-EVs was detected by dynamic light scattering (DLS) with a Nanosizer™ instrument (Malvern Instruments, UK). The EVs were initially diluted in 1 mL PBS solution and were mixed homogeneously. The diluted EVs were then placed into the Nanosight NS300 instrument, and the particle size was automatically monitored and determined according to the Brownian motion and diffusion coefficient. For EV morphology observation, the EV pellet (10 μL) was placed on formvar carbon-coated 200-mesh copper electron microscopy grids, incubated for 5 min at room temperature, and then was allowed to stain with 1% uranyl acetate for 1 min at room temperature. The grid was washed with PBS solution thrice and allowed to semi-dry at room temperature, followed by microscopic observation under a Hitachi H-7650 transmission electron microscope (TEM, Hitachi, Tokyo, Japan). The characterization of EVs was identified by evaluating the expression of specific markers: rabbit antibodies to CD63 (1:2000, ab216130, Abcam, Cambridge, UK), TSG101 (1:10000, ab125011, Abcam), CD81 (1:10000, ab109201, Abcam), CD9 (1:2000, Abcam), Alix (1: 2000, Abcam) and Calnexin (1:100000, ab92573, Abcam) by Western blot analysis.

### Uptake of EVs by NSCLC cells

In order to determine the uptake of MSCs-EVs by A549 and H460 cells, EVs were labeled with a green fluorescent dye (PKH67, Sigma) and 1 × 10^9^ EVs/mL were incubated with A549 and H460 cells at 37 °C for 4 h. The cells were washed with PBS solution for 5 min and were left to fix with 4% paraformaldehyde for 15 min. Nuclei were stained with DAPI fluorescent stain (0.5 mg/mL; Invitrogen, USA) in the dark for 10 min and washed with PBS. Subsequently, the level of green fluorescence in A549 and H460 cells was observed under a fluorescence microscope. In order to detect the migration of miRNA from EVs to A549 and H460 cells, receptor cells were stimulated by MSCs-EVs for 4 h and the expression of miR-598 was analyzed by RT-qPCR. To confirm the transfer of EVs-miR-598, Cy3 labeled miR-598 was used to transfect the MSCs. Then, MSCs expressing cy3-miR-598 were co-cultured with A549 or H460 cells in 24-well Transwell chambers for 48 h. In addition, the A549 and H460 cells were stained with DAPI to microscopically observe the internalization of EVs-miR-598.

### Cell transfection

A549 and H460 cells were transfected with mimic NC, inhibitor NC, miR-598 mimic/inhibitor si-NC, si-THBS2, pcDNA-3.1 and pcDNA-THBS2 according to the instructions provided by the Lipofectamine 3000 Reagent (Invitrogen, USA). The MSCs were treated with the lentiviral vector pLVX-miRNA-598 or the pLVX-inhibitor, which were randomly assigned into the following groups: (1) Paracrine mechanism detection co-culture group; miRNA-598 labeled with Cy3 was used to construct the lentiviral vector: i. Cy3-miR-598 + control; ii. Cy3-miR-598 + GW4869; (2) EVs isolated from MSCs group: i. Inhibitor NC; ii. miR-598 inhibitor; iii. mimic NC; iv. miR-598 mimic. Meanwhile, the A549 and H460 cells were incubated with EVs, and assigned into groups were as follows: (1) sham, (2) MSCs-EVs, (3) MSCs-EVs + inhibitor NC, (4) MSCs-EVs + miR-598 inhibitor, (5) MSCs-EVs + pcDNA-vector, (6) MSCs-EVs + pcDNA-THBS2. All plasmids were purchased from RiboBio Co., Ltd. (Guangzhou, China).

### Cell counting kit-8 assay (CCK-8) assay

CCK-8 (Dojindo, Kyushu Island, Japan) was used to detect the ability of cell proliferation. In brief, after treatment, cells were seeded into 96-well plates with a density of 3 × 10^3^ cells/well. The cells were then incubated according to the following time points; 0, 24, 48, and 72 h. A CCK-8 assay was conducted at every time point by the addition of 10 µL of CCK-8 solution into each well. Following incubation at 37 °C for 2 h, the optical density (OD) was detected using a microplate reader (Molecular Devices, LLC, Sunnyvale, CA, USA) calibrated at a wavelength of 450 nm.

### Transwell assay

Transwell chamber (24-well insert; pore size, 8 μm) (Corning Incorporated, Corning, NY, USA) with or without Matrigel protein mixture (invasion/migration) were used for cell invasion and migration assays, respectively. The transfected cells (6 × 10^3^ cells/well) were seeded into the upper chamber of the 24-well culture inserts coated with a 200 µL serum-free medium. Approximately 400 µL complete medium supplemented with 10% FBS solution was added to the bottom of the inserts, allowing cells to either migrate for 36 h or invade for 48 h. After incubation, the cells on the upper surface of the membrane were removed using cotton swabs, whereas those on the lower filter surfaces were fixed and stained with crystal violet. The number of migrated or invaded cells was counted under the guidance a microscope (IX83; Olympus Corporation, Tokyo, Japan).

### RT-qPCR

Total RNA content was extracted from cultured cells and tissues using a Trizol reagent (Invitrogen, ThermoFisher, Austin, Texas, USA) and was reversely transcribed in order to obtain complementary DNA (cDNA) using Revert Aid first-strand cDNA synthesis kit (Fermentas, Life Sciences, Canada). Afterwards, a RT-qPCR reaction was performed based on the instructions of the SYBR^®^ Premix Ex Taq kit (RR420A, Takara, Japan) in an ABI PRISM^®^ 7900HT System (Takara Biotechnology, Japan). Glyceraldehyde 3-phosphate dehydrogenase (GADPH) was used as the internal control. The relative transcription level of the target gene was calculated using a relative quantitative method (the 2^−△△Ct^ method). The SeraMir EV RNA Purification Kit (System Biosciences, Mountain View, USA) was used to isolate EV miRNA. The cDNA of miRNA was synthesized according to the instructions of the TaqMan microRNA assay kit (Applied Biosystems, Foster City, USA). The RT-qPCR reaction was performed using FastStart Universal SYBR Green Master Mix, (Roche, Indianapolis, USA) including the miRNA-specific forward primer (Sangon Biotech, Shanghai, China) and the universal reverse primer provided by TaqMan microRNA assay kit. The results were normalized using U6 and miR-16. The sequences of the primers used in this study are shown in Supplementary Table 1.

### Western blot analysis

Cells and tissues were lysed using the cell lysis buffer (P0013; Beyotime Biotechnology, Shanghai, China) and incubated at 4 °C for 30 min. Then, the cell lysis buffer was harvested into a 1.5 mL Eppindorf tube for centrifugation at 12,000 g and 4 °C for 15 min. After obtaining the supernatant, the protein concentration was determined by a BCA Protein Assay Kit (Beyotime). The proteins were subsequently separated by 10% sodium dodecyl sulfate-polyacrylamide gel electrophoresis (SDS-PAGE) and then transferred onto a polyvinylidene fluoride membrane (Immobilon P, Millipore, Billerica, USA). The membrane was blocked with 5% skim milk in Tris-buffered saline with 0.1% Tween-20 mixture (TBST) at room temperature for 1 h and later incubated at 4 °C overnight with diluted primary antibodies: Rabbit polyclonal antibodies to Thrombospondin 2 (1:2000, ab84469, Abcam, Cambridge, UK), Rabbit polyclonal antibodies to Ki67 (1:1000, ab15580, Abcam), Rabbit polyclonal antibodies to MMP9 (1:1000, ab38898, Abcam), E-Cadherin (4A2) Mouse mAb (1:1000, #14472, Cell Signaling Technology, CST, Beverly, MA, USA), Vimentin (D21H3) XP® Rabbit mAb (1:1000, #5741, CST), and GAPDH Mouse mAb (1:50000, AC033, Abclonal, China), respectively. The following day, the membrane was washed with TBST mixture 3 times and then incubated with diluted HRP-labeled goat anti-rabbit IgG (1:5000, #7074, CST, USA) or goat anti-mouse IgG (1:5000, #7076, CST, USA) at 37 °C for 1 h. After incubation, the membrane was washed with TBST mixture 3 times; the immunoblots were visualized using enhanced chemiluminescence reagent (Thermo Fisher Scientific, Waltham, USA) and imaged using ChemiDoc XRS Plus luminescent image analyzer (Bio-Rad). Image-Pro Plus 6.0 software (Bio-Rad, Hercules, CA, USA) was used for quantitative protein analysis by measuring the ratio between the gray value of target protein and internal reference (GAPDH). Each experiment was repeated three times.

### Dual luciferase reporter assay

Human THBS2 3′-UTR sequence or the mutant (MUT) sequence of THBS2 3′-UTR, containing the predicted binding sites of miRNA-598, was inserted into the pGL3 promoter vector (Genscript, Nanjing, China). The 293 T cells were seeded in 24-well plates (5 × 10^5^ cells/well) on the day before transfection. Subsequently, the cells were co-transfected with luciferase reporter vectors (0.12 µg) and miR-598 mimic or NC using the Lipofectamine 3000 Reagent (Invitrogen, USA). Luciferase reporter assay was conducted 48 h after transfection according to the manufacturer’s instructions. The Luciferase detection kit (RG005, Beyotime) was used to detect the luciferase activity in a Glomax20/20 fluorescence detector (Promega).

### Nude mouse model with NSCLC

Four-week-old nude male mice (BALB/CJNJU-Foxn1^−/−^ NU/NU) were purchased from the Hunan SJA Laboratory Animal Co., Ltd. In the THBS2 overexpression group, A549 cells with stable THBS2 expression were first constructed. In the EV treatment group, A549 cells (5 × 10^6^ cells) were pretreated with 10 µL of EVs (1 × 10^9^ EVs/mL) for 96 h [34]. Nude mice were randomly divided into several groups of 6 mice, and a 1 mL syringe was used to slowly inoculate 0.2 mL of the suspended cells (1 × 10^7^ cells) into the right subcostal subcutaneous tissues of each nude mouse. After being injected, the mice were allowed free access to food and water. At 3 days after inoculation, the inoculation sites were observed, and the tumor mass sizes were recorded. Here, the lengths of the long (A) and short (B) axes of the subcutaneously implanted tumors were recorded every three days with a Vernier caliper. Tumor volumes were calculated as V = AB^2^/2, and the tumor masses were excised and weighed after the nude mice had been euthanized. Metastasis analysis in vivo: Nude mice were randomly divided into several groups of 15 mice, and 100 μL cell suspension (1 × 10^7^ cells) was injected into each mouse through a tail vein. All the mice were euthanized after 6 weeks post-inoculation, and the number of metastatic nodules in the lungs were counted during autopsy. The lung tissues were then fixed in 10% neutral PB-buffered formalin, embedded in paraffin, and stained with hematoxylin-eosin (H&E) (Solaibao, G1120) to visualize the morphology of metastatic nodules in the lungs of mice.

### Statistical analysis

All experimental data were analyzed using the SPSS 21.0 software (IBM Corp. Armonk, NY, USA). Measurement data were summarized by the mean ± standard deviation from at least three independent experiments. A comparison between tumor tissues and normal tissues was performed by a paired t-test, while comparisons between other two groups were performed by an unpaired t-test. Comparisons among multiple groups were performed by one-way analysis of variance (ANOVA) with Tukey’s post hoc test. Comparison among groups at different time points was performed using repeated measures of ANOVA with Bonferroni’s post hoc test. *p* < 0.05 indicated the difference was statistically significant.

## Supplementary information

Supplementary information

## Data Availability

All of the data and materials are available from the corresponding author upon reasonable request.
