# Supplementary material for: Mesenchymal stem cell-derived extracellular vesicles transfer miR-598 to inhibit the growth and metastasis of non-small-cell lung cancer by targeting THBS2
Source: Cell Death Discov. 2023 Jan 6;9:3. doi: 10.1038/s41420-022-01283-z (PMC9822924; doi:10.1038/s41420-022-01283-z)
Supplement: Supplementary file 1 — Supplementary information [file 41420_2022_1283_MOESM1_ESM.docx]

**
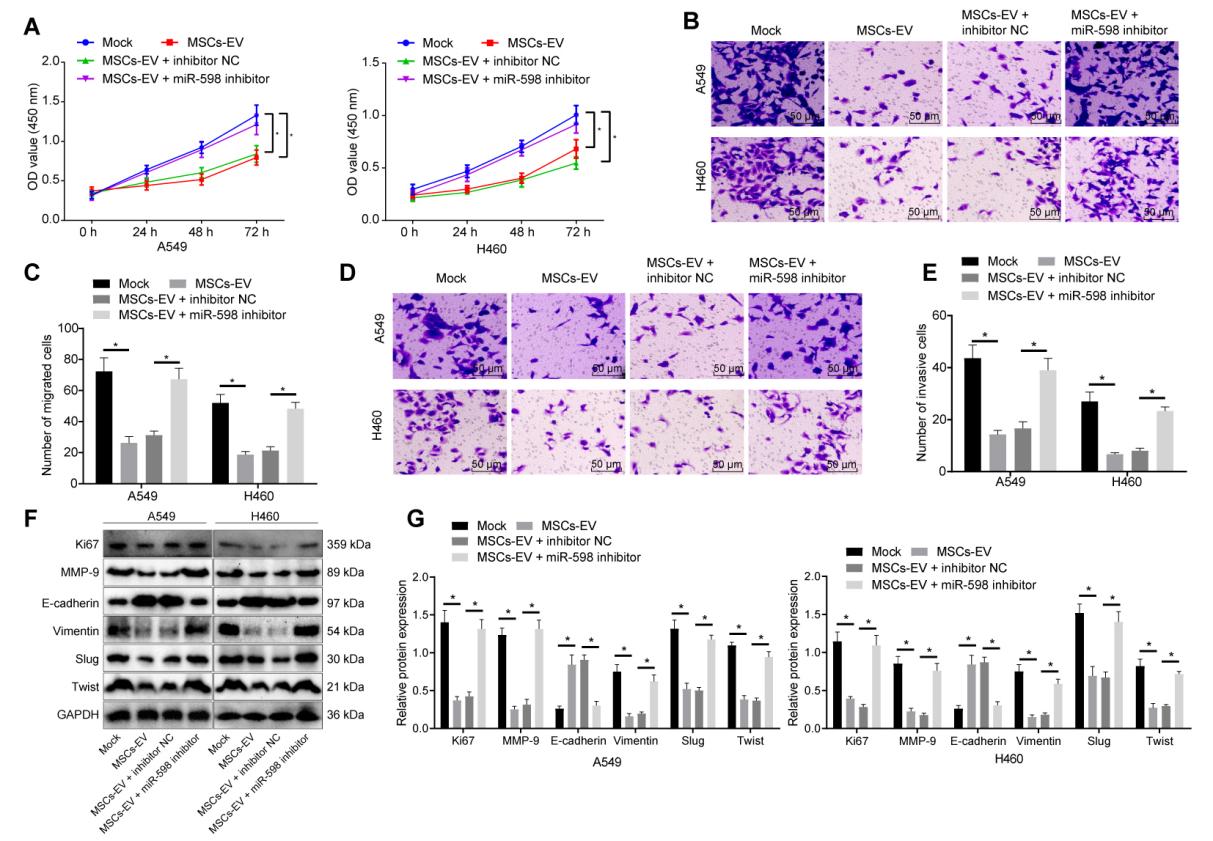
**

**Fig. 3**

Effects of EVs-derived miR-598 on the proliferation, migration, and invasion abilities of NSCLC cells.

**A** Proliferative ability of A549 and H460 cells detected by CCK-8 assay. **B** Migrated ability of A549 and H460 cells detected by Transwell assay. **C** Statistical analysis of (**B**). **D** Invasive ability of A549 and H460 cells detected by Transwell assay. **E** Statistical analysis of (**D**). **F** Protein bands of Ki67, E-cadherin, Vimentin, MMP-9, Slug, and Twist by Western blot analysis. **G** Protein levels of Ki67, E-cadherin, Vimentin, MMP-9, Slug, and Twist by Western blot analysis (statistical analysis of (**F**)). *p < 0.05 between two groups. Data between two groups were compared by an unpaired t test. Data among multiple groups were compared by ANOVA with Tukey’s post hoc test. Comparisons among groups at different time points were performed using repeated measures ANOVA with Bonferroni’s post hoc test. Cell experiments were repeated three times.

**Supplementary Table 1.** Primer sequences used for RT-qPCR

| Target Gene | Primer sequences |
| --- | --- |
| miR-598 | F: 5’-GCGGTGATCCCGATGGTGUGAGC-3’ |
|  | R: universal primer’ |
| U6 | F:5’- CTCGCTTCGGCAGCACATA-3’ |
|  | R: universal primer |
| miR-16 | F: 5’-CCAGTAUUAACTGTGCTGCTGA-3’ |
|  | F: universal primer |
| THBS2 | F: 5’-GACACGCTGGATCTCACCTAC-3’ |
|  | R: 5’-GAAGCTGTCTATGAGGTCGCA-3’ |
| GAPDH | F: 5’-GGAGCGAGATCCCTCCAAAAT-3’ |
|  | R: 5’-GGCTGTTGTCATACTTCTCATGG-3’ |
| Ki67 | F: 5’-ACGCCTGGTTACTATCAAAAGG-3’ |
|  | R: 5’-CAGACCCATTTACTTGTGTTGGA-3’ |
| MMP-9 | F: 5’-TGTACCGCTATGGTTACACTCG-3’ |
|  | R: 5’-GGCAGGGACAGTTGCTTCT-3’ |
| E-cadherin | F: 5’-CGAGAGCTACACGTTCACGG-3’ |
|  | R: 5’-GGGTGTCGAGGGAAAAATAGG-3’ |
| Vimentin | F: 5’-GACGCCATCAACACCGAGTT-3’ |
|  | R: 5’-CTTTGTCGTTGGTTAGCTGGT-3’ |

**Notes:** RT-qPCR, reverse transcription quantitative polymerase chain reaction; F, forward; R, reverse; miR-598, microRNA 598; U6, U6 small nuclear RNA; miR-16, microRNA-16; THBS2, Thrombospondin 2; GAPDH, Glyceraldehyde-3-phosphate dehydrogenase; Ki67 Proliferation Marker Protein Ki-67; MMP-9, Matrix Metallopeptidase 9; E-cadherin, Epithelial Cadherin.


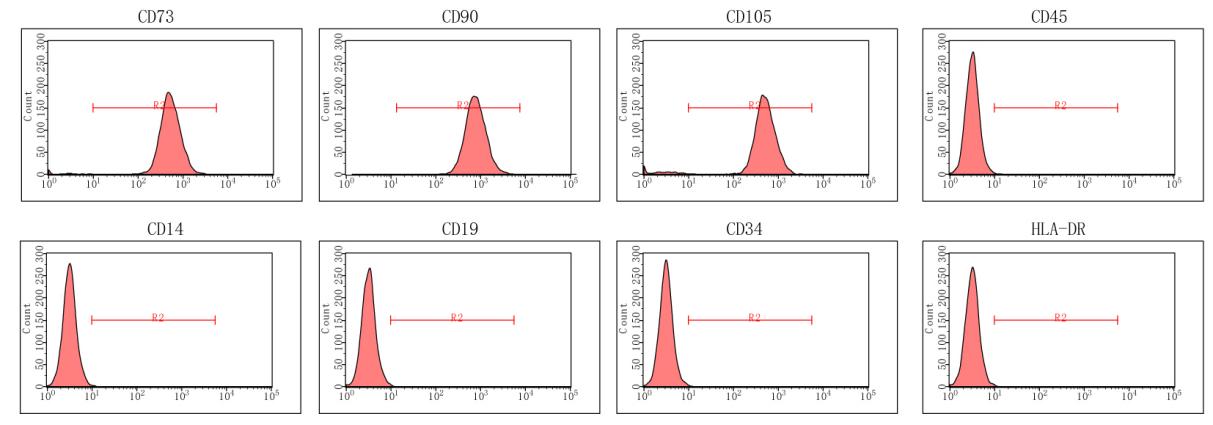


**Supplementary Fig. 1** Expression of surface antigens of MSCs detected by flow cytometry


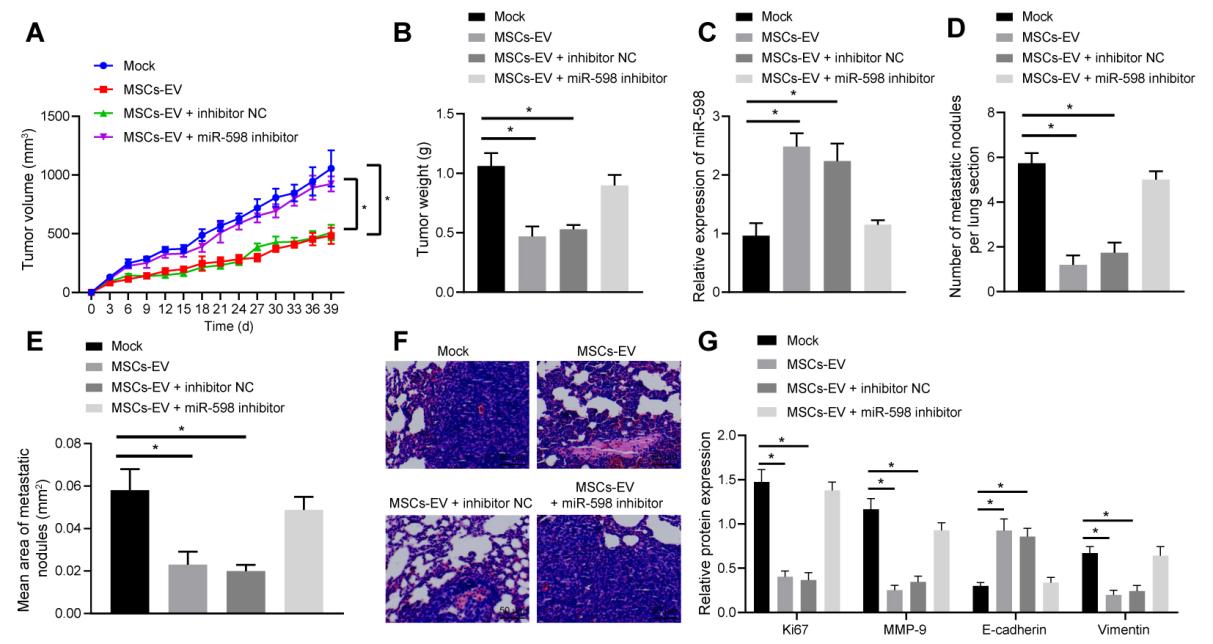


**Supplementary Fig. 2** Effects of EVs-derived miR-598 on tumor growth and metastasis of NSCLC in vivo. **A**. Tumor volume in the nude mice after different treatments. **B**. Tumor weight in the nude mice. **C**. Expression of miR-598 in tumors of the nude mice measured by RT-qPCR. **D**. Number of metastatic nodules in the lungs of nude mice after different treatment. **E**. Area of metastatic nodules in the lungs of nude mice. **F**. Morphology of metastatic nodules in the lungs of nude mice of observed by H&E staining. **G**. Protein levels of Ki67, E-cadherin, Vimentin, and MMP-9 in metastatic nodules in the lungs of nude mice by Western blot analysis. n = 6 in each group in the subcutaneous tumorigenesis experiments, n = 15 in each group in the pulmonary metastasis experiments. * p < 0.05 between two groups. Data among multiple groups were compared by ANOVA with Tukey’s post hoc test. Comparisons among groups at different time points were performed using repeated measures ANOVA with Bonferroni’s post hoc test.
